# Association among abnormal glycolipids, reproductive hormones, and cognitive dysfunction in female patients with bipolar disorder

**DOI:** 10.1186/s12888-024-05831-y

**Published:** 2024-05-21

**Authors:** Tianxiang Zou, Min Yang, Zhuohui Chen, Haiqing Xie, Jing Huang, Yue Qin, Furu Liu, Haiyu Chen, Xuelei Xu, Jindong Chen, Hui Tang, Hui Xiang, Haishan Wu, MingHui Liu, Wenbo Luo, Jieyu Liu, Ziwei Teng

**Affiliations:** 1 Department of Psychiatry, National Clinical Research Center for Mental Disorders, and National Center for Mental Disorders, The Second Xiangya Hospital of Central South University, Changsha, 410011 Hunan China; 2 Department of Ultrasound Diagnosis, The Second Xiangya Hospital of Central South University, Changsha, 410011 Hunan China; 3 Department of Neurosurgery, Xiangya Hospital, Central South University, 87 Xiangya Road, Changsha, 410008 China

**Keywords:** Female, Bipolar disorder, Glycolipid level, Reproductive hormone, Cognitive dysfunction

## Abstract

**Background:**

Patients with bipolar disorder (BD) show abnormalities in glucolipid metabolism and reproductive hormone levels, which are of concern in women with BD. This study was dedicated to investigating the glucolipid and reproductive hormone levels of female patients, and to preliminarily investigating their relationships with cognition.

**Methods:**

A total of 58 unmedicated female BD patients, 61 stable-medicated female BD patients, and 63 healthy controls (HC) were recruited in this study. Serum glycolipid indexes and reproductive hormones were measured. Cognitive function was assessed using the Repeatable Battery for the Assessment of Neuropsychological Status (RBANS) and the Stroop Color-Word Test (Stroop test).

**Results:**

Patients with BD showed significant cognitive impairment (*p* < 0.05), which was not affected by medication. Triglycerides (TG), luteinizing hormone (LH), and high-density lipoprotein cholesterol (HDL-c) were altered in stable-medicated BD patients. In addition, regression analysis showed that progesterone (PRGE) and prolactin (PRL) were negatively associated with cognitive performance in stable-medicated BD patients.

**Conclusions:**

Female BD patients may have cognitive deficits and abnormal levels of glycolipids and reproductive hormones. And abnormal levels of glycolipids and reproductive hormones may be associated with cognitive dysfunction in female BD patients.

**Supplementary Information:**

The online version contains supplementary material available at 10.1186/s12888-024-05831-y.

## Background

Bipolar disorder (BD) is a chronic and recurrent serious mental illness characterized by alternating episodes of depression and mania (bipolar I disorder) or hypomania (bipolar II disorder), with a lifetime prevalence estimated at 1.0% worldwide [1]. The latest National Survey of Mental Disorders showed that the lifetime prevalence of BD in the general population in China is 0.6% (95% CI: 0.4–0.7%) [2].

In the past decade, the importance of cognitive impairment associated with BD has begun to be recognized. Compared to healthy controls, patients with BD performed worse in the areas of attention, executive functioning, processing speed, working memory, and verbal memory, even at remission [3, 4]. Cognitive impairment may help explain the impairment in psychosocial functioning in patients with BD [5]. And cognitive impairment has also been recognized as one of the core clinical manifestations of BD [6]. One study suggested that there was gender difference in the biomarkers of BD, and these biomarkers were associated with cognitive and psychosocial functioning [7]. In this study 7, high C-reactive protein levels were associated with impaired cognitive functioning in female BD patients, whereas in male BD patients, both high C-reactive protein and homocysteine levels were associated with poorer psychosocial functioning. Moreover, only C-reactive protein could serve as a predictor of cognitive performance in female BD patients, which was not observed in male BD patients 7. Therefore, exploring the biomarkers related to cognitive function may achieve the purpose of preventing and reversing cognitive impairment in female BD patients.

Hormones of the hypothalamic-pituitary-gonadal (HPG) axis not only regulate reproductive functions, but also regulate cognitive functions by binding to corresponding receptors in the human brain [8, 9]. The most studied of these hormones are the reproductive steroids, and the effects of estrogens, progestins, and androgens in the brain are related to development, maintenance, and cognitive functions [10]. The study of Xie et al. suggested that a reactivated HPG axis and elevated PRL levels can influence brain activity, and that such effects may underlie neuroendocrine changes in mood, cognition, and social behavior in early adolescent girls [11]. It was found that in patients with early psychosis, higher prolactin levels were associated with slower processing speed, and prolactin may be involved in the pathological process of cognitive impairment in patients with BD [12, 13]. Many clinical studies have shown that a progesterone (PRGE) receptor antagonist mifepristone could promote cognitive improvement in BD patients [14, 15]. Hormonal or steroid-targeting drugs associated with the HPA axis may help to improve cognitive function in female BD patients [16].

The prevalence and incidence of hyperlipidemia have been reported to be higher in patients with BD than in general population [17]. It has been shown that altered metabolic indexes are associated with neurocognitive decline in BD patients. One study reported that in drug-naïve BD patients, elevated triglyceride (TG) level was correlated with cognitive deficits in attention and delayed memory, and elevated fasting blood glucose (FBG) level was correlated with cognitive deficits in language [18]. Another study found that decreased high density lipoprotein (HDL) level in BD patients was correlated with cognitive deficits in immediate memory and language [19]. It was also found that persistently high levels of glucose can lead to insulin resistance and thus affect cognitive function [20]. Moreover, hyperglycemia and hyperinsulinemia affect cognitive function through pathways associated with neuroplasticity [21, 22]. These findings suggest that serum glucose, insulin and lipids are associated cognitive function.

This study aimed to investigate serum glucose, lipids, reproductive hormone levels, and cognitive function in unmedicated and stable-medicated female BD patients, and to investigate the association among routine glucose and lipid metabolic markers, serum reproductive hormone levels and cognitive impairment in female BD patients.

## Methods

### Participants

A total of 58 unmedicated BD outpatients and 61 stable-medicated BD outpatients from the Second Xiangya Hospital were recruited between December 2019 and September 2021. The study protocol was approved by the ethics committee of the Second Xiangya Hospital of Central South University, Changsha, China. All participants provided written informed consent before the start of the study and met the following inclusion criteria: (1) age 16–45 years, female Chinese; (2) BD diagnosis without other psychiatric disorders using the Diagnostic and Statistical Manual of Mental Disorders, Fifth Edition (DSM-5) by two psychiatrists; (3) no previous and current use of any psychotropic drugs or stable psychotropic medication regimen for ≥ 6 months; (4) participants voluntarily participated in the study and signed informed consent and were able to comply with laboratory tests, and other study procedures (If the participant was a minor, the informed consent form was signed by the participant’s guardian). The exclusion criteria were: (1) presence of any other medical disease affecting reproductive endocrine function; (2) taking contraceptives, immunosuppressants, and other drugs that may affect reproductive function within six months; (3) currently pregnant, breastfeeding or planning to become pregnant; (4) in perimenopause or post menopause; (5) with obvious suicidal tendencies; (6) serious neurological disease with a clear family history or underlying risk; (7) comorbid other severe acute or chronic diseases, mental diseases or abnormal laboratory tests with clinical significance; (8) judged by the investigator to be unsuitable to participate in this study.

During the same period, 63 female Chinese healthy controls (HCs) were recruited from the local community. They were assessed by using DSM-5 to rule out the presence of any mental disorder.

### Demographic and clinical assessment

Age and years of education were collected from female participants after they signed a consent form. We measured height and weight using a calibrated measuring instrument to calculate body mass index [BMI = weight (kg)/ height (m^2^)]. For participants with BD, the duration of disease and age at first onset were also collected. Manic symptoms were assessed using the Young Mania Rating Scale (YMRS) [23], depressive symptoms using the Hamilton Depression Scale (HAMD) [24], and anxiety symptoms using the Hamilton Anxiety Inventory (HAMA) [25] (based on the patient’s past week’s performance), respectively. Each patient was initially diagnosed by at least one outpatient or resident psychiatrist (Associate professor or higher). A diagnostic review was then conducted by two consistently trained psychiatrists using the MINI-International Neuropsychiatric Interview (M.I.N.I.).

Participants in the HC group were screened by their past medical history and an interview-based psychiatric examination to exclude patients with psychiatric disorders.

### Cognitive assessment

The Repeatable Battery for the Assessment of Neuropsychological Status (RBANS) [26] was used to assess cognitive function of all participants. The RBANS contains 12 subtests which can be summarized into five age-adjusted indexes, including immediate memory (list learning and story memory subtests), visuospatial/constructional (line orientation and figure copy subtests), language (picture naming and semantic fluency subtests), attention (digit span and coding subtests), and delayed memory (list recall, story recall, figure recall, and list recognition subtests). The Chinese version of RBANS used in this study has good reliability and validity and can be performed in 30 min [27]. Before evaluation, we had educated and mocked trials for the participants to be familiar with the RBANS.

The Stroop color-word test (Stroop test) mainly measures perceptual conversion, selective attention and inhibition of habitual response patterns, and is more sensitive to psychological control and response plasticity in executive functions [28]. The Chinese version of the Stroop test consisting of 3 subtests (word, color and word interference) was used in this study. Each subtest is limited to 45 seconds. The more the participants read the correct number, the higher the scores are. The total scores of the Stroop test indicate cognitive flexibility and inhibition ability.

### Serum glycolipid and hormone levels measurements

To limit possible biases, blood samples from all participants were collected between 8 a.m. and 10 a.m. on days 2–5 of their menstrual cycle after overnight fasting. Since hormone levels are relatively stable during this period, it helps to provide a more precise and accurate assessment of ovarian function. The blood samples were clotted at room temperature for 40 min and then centrifuged at 3000 rpm for 10 min. Serum samples were separated and stored at -80 Celsius before use.

The levels of TG, cholesterol (CHOL), low-density lipoprotein cholesterol (LDL-c), HDL-c, FBG, insulin, follicle-stimulating hormone(FSH), prolactin (PRL), estradiol (E2), testosterone II (TST II), PRGE and anti-Müllerianhormone (AMH) were measured by technicians who were unaware of the clinical conditions of all participants, using an automatic biochemical analysis system (Abbott c16000 instrument). Insulin resistance index (IRI) was calculated by using the formula of homeostasis model assessment of insulin resistance (HOMA-IR): fasting serum insulin (mIU/mL) × FPG (mg/dL)/22.5.

### Data analysis

All statistical analyses were performed in SPSS (IBM Corp, Armonk, NY; Version 26) with a significant level of two-tailed *p*-values of 0.05. Continuous variables were shown as mean ± SD. Categorical variables were shown as N1:N2. Kolmogorov–Smirnov one-sample test was used to measure the normal distribution of continuous variables. Categorical variables were tested by Chi-square test, and continuous variables were tested by analysis of variance (ANOVA) or Kruskal-Wallis H test. Bonferroni correction was used for pairwise post-hoc test.

Pearson correlation analysis was used to explore the correlation between cognition and serum glucose/lipid/reproductive hormone levels in each group. Partial correlation analysis was used to explore the correlation between cognition and reproductive hormone levels by controlling for glucose and lipid levels which were correlated with reproductive hormone levels. Further stepwise multivariate regression analysis was used to evaluate the contributions of serum glucose, lipids, and reproductive hormones to cognition in the unmedicated BD outpatients, stable-medicated BD outpatients and all BD outpatients.

### The patient and public involvement statement

Patients or the public were not involved in the design, or conduct, or reporting, or dissemination plans of our research.

## Results

### Demographic and clinical characteristics

Table 1 shows the clinical variables and demographic characteristics of three groups, including 58 unmedicated patients with BD, 61 stable-medicated BD patients, and 63 HCs. There was no significant difference among the three groups in age (F = 0.858, *p* = 0.426), education (F = 2.794, *p* = 0.064), BMI (F = 2.858, *p* = 0.060), smoking history (χ2 = 2.434, *p* = 0.296 and drinking history (χ2 = 1.503, *p* = 0.472). However, significant differences were found in HDRS, HAMA, and YMRS scores, onset age of illness, and duration of illness (*p < 0.05*) between unmedicated and stable-medicated BD patients.

**Table 1:** Demographic and clinical data of all participants

	Unmedicated BD (*n* = 58)	Medicated BD(*n* = 61)	HC (*n* = 63)	F/t/χ^2^	*p* value	Adjusted *p* value
Age (year)	22.62 ± 5.38	23.89 ± 6.00	23.48 ± 4.64	0.858	0.426^a^	/
Education (year)	15.45 ± 2.05	15.15 ± 2.21	16.16 ± 2.96	2.794	0.064^a^	/
BMI	22.20 ± 3.35	23.10 ± 3.41	22.38 ± 3.05	2.858	0.060^a^	/
History of drinking(yes: no)	6:52	3:58	5:43	1.503	0.472^b^	/
History of smoking(yes: no)	9:49	6:55	3:45	2.434	0.296^b^	/
HAMD	22.17 ± 6.15	13.10 ± 8.13	/	6.838	<0.001^c**^	/
HAMA	27.12 ± 7.34	16.00 ± 10.06	/	6.861	<0.001^c**^	/
YMRS	13.78 ± 7.41	21.68 ± 7.49	/	3.208	0.002^c**^	/
Onset age of illness (year)	20.36 ± 5.31	9.77 ± 6.18	/	2.320	0.022^c*^	/
Psychotropic medication (n)						
Valproate	/	37	/	/	/	/
Other mood stabilizers	/	24	/	/	/	/
Duration of illness (year)	1.96 ± 2.28	5.57 ± 3.65	/	6.423	<0.001^c**^	/

Adjusted *p* value: *p* value after Bonferroni correction. BD, bipolar disorder; HC, healthy controls; BMI, body mass index; HAMD, the Hamilton Depression Scale; HAMA, the Hamilton Anxiety Inventory; YMRS, the Young Mania Rating Scale. (a) Analysis of variance. (b) χ^2^ test. (c) Independent sample t test

* p<0.05

** p<0.01

### Cognitive performance

Table 2 shows the comparison of cognitive domains among the three groups, with significant differences in immediate memory (F = 28.428, *p* < 0.001), visuospatial/constructional (F = 9.426, *p* < 0.001), language (F = 26.513, *p* < 0.001), attention (F = 16.771, *p* < 0.001), delayed memory (F = 17.714, *p* < 0.001), RBANS’ total scores (F = 40.340, *p* < 0.001), Stroop word (F = 34.204, *p* < 0.001), Stroop color (F = 28.063, *p* < 0.001), Stroop word-color (F = 22.099, *p* < 0.001) and Stroop total scores (F = 39.723, *p* < 0.001).

**Table 2:** Comparison of cognitive function among three groups

	a Unmedicated BD (*n* = 58)	b Medicated BD (*n* = 61)	c HC(*n* = 63)		F	*p* value	Adjusted *p* value
Cognitive assessment							
Immediate memory	38.64 ± 8.72	38.90 ± 8.92	47.95 ± 5.43	28.428		<0.001^**^	a< c, *p*<0.001^**^ b< c, *p*<0.001^**^
Visuospatial/ Constructional	33.79 ± 3.83	34.15 ± 2.48	36.05 ± 2.87	9.426		<0.001^**^	a< c, *p*<0.001^**^ b< c, *p* = 0.002^**^
Language	28.20 ± 4.73	28.24 ± 4.98	33.52 ± 4.30	26.513		<0.001^**^	a< c, *p*<0.001^**^ b< c, *p*<0.001^**^
Attention	69.29 ± 10.30	69.51 ± 9.92	77.87 ± 7.87	16.771		<0.001^**^	a< c, *p*<0.001^**^ b< c, *p*<0.001^**^
Delayed memory	47.33 ± 6.35	46.10 ± 10.28	53.56 ± 4.71	17.714		<0.001^**^	a< c, *p*<0.001^**^ b< c, *p*<0.001^**^
RBANS total	217.25 ± 25.05	216.89 ± 24.71	248.95 ± 18.19	40.340		<0.001^**^	a< c, *p*<0.001^**^ b< c, *p*<0.001^**^
Stroop word	100.00 ± 10.80	93.56 ± 17.24	117.83 ± 16.77	34.204		<0.001^**^	a< c, *p*<0.001^**^ b< c, *p*<0.001^**^
Stroop color	69.05 ± 15.70	64 0.30 ± 14.14	82.92 ± 13.34	28.063		<0.001^**^	a< c, *p*<0.001^**^ b< c, *p*<0.001^**^
Stroop color-word	38.09 ± 10.78	38.07 ± 9.74	48.70 ± 10.25	22.099		<0.001^**^	a< c, *p*<0.001^**^ b< c, *p*<0.001^**^
Stroop total	207.14 ± 37.92	195.92 ± 35.17	249.44 ± 32.78	39.723		<0.001^**^	a< c, *p*<0.001^**^ b< c, *p*<0.001^**^

Adjusted *p* value: *p* value after Bonferroni correction. BD, bipolar disorder; HC, healthy controls; RBANS, the Repeatable Battery for the Assessment of Neuropsychological Status

* *p*<0.05

** *p*<0.01

After the Bonferroni post-hoc test, outcomes revealed that immediate memory, visuospatial/constructional, language, attention, delayed memory, RBANS’ total scores, Stroop word, Stroop color, Stroop color-word, and Stroop total scores were significantly lower in BD patients than in controls (*p* < 0.05). However, no significant difference was observed in all cognitive domains between the unmedicated and stable-medicated patients.

### Serum glycolipid and hormone levels

Table 3 shows the comparison of glycolipids and reproductive hormones among the three groups. Only 48 of the 63 subjects in the healthy control group had blood samples collected, and 15 were unwilling to accept the request for blood samples. There was no significant difference among the three groups in FSH, PRL, E2, TST II, PRGE, AMH, FBG, CHOL, LDL-c, insulin, and IRI. However, significant differences were found among three groups in LH (F = 4.418, *p* = 0.014), TG (F = 4.859, *p* = 0.009), HDL-c (F = 5.844, *p* = 0.004) and HDL-c/CHOL (F = 6.471, *p* = 0.002).

**Table 3:** Comparison of serum reproductive hormone and glycolipid levels among three groups

	a	b	c	F	*p* value	Adjusted
	Unmedicated BD (n = 58)	Medicated BD (n = 61)	HCs(n = 48)			*p* value
Reproductive hormone and glycolipid levels						
LH (IU/L)	4.46 ± 2.13	5.90 ± 5.23	4.01 ± 1.69	4.418	0.014^*^	c<b, *p* = 0.019*
FSH (IU/L)	6.27 ± 1.69	6.33 ± 1.60	6.84 ± 1.63	1.832	0.163	/
PRL (µg/L)	11.51 ± 8.94	12.97 ± 12.02	12.76 ± 8.86	0.347	0.708	/
E2 (nmol/L)	0.18 ± 0.11	0.17 ± 0.07	0.17 ± 0.08	0.42	0.658	/
TST II (nmol/L)	0.94 ± 0.33	0.97 ± 0.49	0.96 ± 0.23	0.077	0.926	/
PRGE (µg/L)	0.70 ± 0.32	1.13 ± 2.85	0.71 ± 0.26	1.166	0.314	/
AMH (ng/mL)	4.32 ± 2.84	4.73 ± 2.69	3.66 ± 1.99	2.352	0.098	/
TG (mmol/L)	1.00 ± 0.80	1.31 ± 1.5	0.80 ± 0.38	4.859	0.009^**^	c<b, *p* = 0.008**
CHOL (mmol/L)	4.21 ± 0.75	4.23 ± 0.72	4.12 ± 0.58	0.423	0.656	/
HDL-c (mmol/L)	1.37 ± 0.29	1.22 ± 0.27	1.34 ± 0.21	5.844	0.004^**^	b<a, *p* = 0.004**
LDL-c (mmol/L)	2.46 ± 0.68	2.60 ± 0.70	2.42 ± 0.50)	1.194	0.306	/
HDL-c/CHOL	0.33 ± 0.07	0.29 ± 0.07	0.33 ± 0.05	6.471	0.002^**^	b<a, *p* = 0.005**
						b<c, *p* = 0.012*
FBG (mmol/L)	4.60 ± 0.65	4.55 ± 0.46	4.63 ± 0.58	0.273	0.762	/
Insulin (mIU/L)	10.42 ± 5.55	11.73 ± 8.73	9.10 ± 4.21	2.139	0.121	/
IRI	2.18 ± 1.45	2.41 ± 2.08	2.01 ± 1.02	0.856	0.427	/

Adjusted *p* value: *p* value after Bonferroni correction. BD, bipolar disorder; HC, healthy controls; LH, luteinizing hormone; FSH, follicle-stimulating hormone; PRL, prolactin; E2, estradiol; TST II, testosterone II; PRGE, progesterone; AMH, anti-Müllerian hormone; TG, triglyceride; CHOL, cholesterol; HDL-c, high-density lipoprotein cholesterol; LDL-c, low-density lipoprotein cholesterol; FBG, fasting blood glucose; IRI, Insulin Resistance Index

*p<0.05

** *p*<0.01

Bonferroni post-hoc test revealed that LH and TG in the stable-medicated BD patients were significantly higher than in controls (*p* < 0.05). And HDL-c in the stable-medicated BD patients was significantly lower than in the unmedicated BD patients (*p* < 0.01). HDL-c/CHOL in the stable-medicated BD patients was significantly lower than in the unmedicated BD patients and controls (*p* < 0.01).

### Correlation analyses of glycolipids, reproductive hormones, and cognitive variables

Table 4 presents the results of correlation analyses in the unmedicated BD patients. The PRL was positively correlated with attention (*r* = 0.272, *p* < 0.05). And TG was positively correlated with language (*r* = 0.336, *p* < 0.01). The level of LDL-c was negatively correlated with Stroop color (*r* = -0.355, *p* < 0.01), Stroop word-color (*r* = -0.276, *p* < 0.05) and Stroop total scores (*r* = -0.312, *p* < 0.05). HDL-c/CHOL was positively correlated with Stroop color (*r* = 0.278, *p* < 0.05) and Stroop total scores (*r* = 0.277, *p* < 0.05). After controlling for HDL-c level which was correlated with TST II (*r* = 0.294, *p* < 0.05), a correlation between PRL and attention (*r* = 0.278, *p* < 0.05) remained significant.

**Table 4:** Correlation of serum hormones, glycolipid and cognitive function in unmedicated BD patients

BD (*n* = 58)	LH	FSH	PRL	E2	TST II	PRGE	AMH	TG	CHOL	HDL-c	LDL-c	HDL-c/CHOL	FBG	Insulin	IRI	
	*r*															
Immediate memory	-0.101	-0.057	-0.088	0.072	-0.080	-0.054	0.061	0.121	-0.191	0.012	-0.215	0.134	0.090	-0.025	0.005	
Visuospatial/ Constructional	0.035	-0.082	0.000	0.100	0.012	0.076	0.187	0.064	0.023	0.083	-0.007	0.004	-0.110	-0.044	-0.066	
Language	-0.075	-0.002	-0.012	0.005	-0.186	-0.140	0.225	0.336^**^	0.075	-0.177	0.115	-0.237	0.126	-0.022	0.049	
Attention	-0.004	0.020	0.272^*^	-0.088	-0.102	0.094	0.076	-0.184	-0.183	0.013	-0.171	0.139	-0.065	-0.060	-0.100	
Delayed memory	0.069	0.137	-0.002	0.043	-0.223	-0.142	0.208	0.205	-0.053	-0.170	0.034	-0.131	-0.020	-0.002	0.011	
RBANS total	-0.028	0.010	0.078	0.016	-0.159	-0.031	0.176	0.092	-0.138	-0.054	-0.116	0.026	0.006	-0.045	-0.037	
Stroop word	0.003	-0.082	0.157	-0.116	0.111	0.146	0.223	0.006	-0.100	0.153	-0.195	0.212	-0.112	-0.298^*^	-0.287^*^	
Stroop color	0.102	0.020	-0.034	0.013	0.002	-0.041	0.224	-0.026	-0.247	0.142	-0.355^**^	0.287^*^	-0.021	-0.149	-0.146	
Stroop color-word	-0.083	0.000	0.006	-0.074	-0.096	0.163	0.146	-0.073	-0.231	0.069	-0.276^*^	0.226	-0.044	-0.130	-0.131	
Stroop total	0.020	-0.028	0.057	-0.067	0.023	0.094	0.233	-0.029	-0.212	0.146	-0.312^*^	0.277^*^	-0.071	-0.231	-0.225	

* *p*<0.05

** *p*<0.01

Table 5 presents the results of correlation analyses in the stable-medicated BD patients. PRL was negatively correlated with attention (*r* = -0.373, *p* < 0.01) and Stroop word-color (*r* = -0.272, *p* < 0.05). TST II was positively correlated with immediate memory (*r* = 0.274, *p* < 0.05). PRGE was negatively correlated with attention (*r* = -0.299, *p* < 0.05) and Stroop word-color (*r* = -0.281, *p* < 0.05). After controlling for FBG which was correlated with FSH (*r* = 0.260, *p* < 0.05)) and HDL-c/CHOL which was correlated with PRGE (*r* = 0.296, *p* < 0.05), PRL was correlated with attention (*r* = -0.394, *p* < 0.05), RBANS’ total score (*r* = -0.290, *p* < 0.05) and Stroop word-color (*r* = -0.262, *p* < 0.05), TST II was correlated with immediate memory (*r* = -0.284, *p* < 0.05) and PRGE was correlated with Stroop word-color (*r* = -0.325, *p* < 0.05).

**Table 5:** Correlation of serum hormones, glycolipid levels and cognitive function in stable-medicated BD patients

BD (*n* = 61)	LH	FSH	PRL	E2	TST II	PRGE	AMH	TG		CHOL	HDL-c	LDL-c	HDL-c/CHOL	FBG	Insulin	IRI	
									*r*								
Immediate memory	-0.112	0.014	-0.075	0.019	0.274^*^	0.128	-0.194	0.051		0.018	0.028	0.010	0.004	-0.183	-0.016	-0.048	
Visuospatial/ Constructional	0.046	-0.016	-0.017	-0.146	0.029	-0.299^*^	0.065	0.134		0.007	-0.007	-0.040	-0.044	-0.141	-0.179	-0.222	
Language	-0.076	0.136	-0.011	0.098	-0.088	-0.092	-0.100	-0.129		-0.126	0.057	-0.111	0.116	-0.121	-0.219	-0.230	
Attention	-0.080	-0.147	-0.373^**^	-0.145	-0.133	-0.242	-0154	-0.036		-0.071	-0.020	-0.079	-0.008	− 0.0054	-0.176	-0.190	
Delayed memory	0.025	0.003	-0.128	0.056	-0.095	0.087	-0.052	0.069		0.178	0.028	0.172	-0.124	-0.161	0.094	0.064	
RBANS total	-0.073	-0.027	-0.234	-0.023	-0.206	-0.026	-0.167	0.020		0.027	0.024	0.017	-0.034	-0.193	-0.099	-0.136	
Stroop word	-0.110	0.083	0.041	-0.248	-0.022	-0.057	-0.028	0.109		-0.111	-0.019	-0.124	0.002	-0.203	-0.169	-0.207	
Stroop color	-0.105	0.064	-0.120	-0.088	-0.001	-0.206	-0.073	-0.091		-0.107	0.015	-0.072	0.034	0.026	-0.161	-0.162	
Stroop color-word	0.032	-0.007	-0.272^*^	-0.023	-0.049	-0.281^*^	-0.070	-0.188		-0.003	0.124	0.020	0.097	0.029	-0.119	-0.100	
Stroop total	-0.088	0.065	-0.103	-0.163	-0.025	-0.189	-0.063	-0.035		-0.098	0.031	-0.084	0.042	0-0.081	-0.181	-0.194	

* *p*<0.05

** *p*<0.01

Table 6 presents the results of correlation analyses in the HC group. HDL-c was positively correlated with immediate memory (*r* = 0.313, *p* < 0.05) and delayed memory(*r* = 0.295, *p* < 0.05). And HDL-c/CHOL was positively correlated with delayed memory (r = 0.324, *p* < 0.05).

**Table 6:** Correlation of serum hormones, glycolipid levels and cognitive function in HCs

BD (*n* = 63)	LH	FSH	PRL	E2	TST II	PRGE	AMH	TG		CHOL	HDL-c	LDL-c	HDL-c/CHOL	FBG	Insulin	IRI	
									*r*								
Immediate memory	-0.035	-0.055	0.092	0.114	0.040	0.076	-0.073	0.101		0.063	0.313*	-0.018	0.238	0.079	0.063	0.062	
Visuospatial/ Constructional	-0.050	-0.065	0.081	0.143	0.220	0.065	0.034	0.121		-0.014	0.132	-0.077	0.160	-0.005	0.008	0.086	
Language	0.003	-0.118	0.135	-0.071	-0.204	-0.099	-0.107	0.190		0.217	0.265	0.148	0.020	0.147	-0.026	-0.049	
Attention	0.010	0.196	0.193	0.142	-0.092	-0.084	0.203	0.012		-0.053	-0.003	0.011	0.006	0.052	0.075	0.095	
Delayed memory	-0.032	-0.026	0.198	0.121	0.034	0.047	-0.155	0.052		-0.097	0.295*	-0.144	0.324*	0-0.111	-0.001	0.023	
RBANS total	-0.019	0.025	0.205	0.128	-0.041	-0.019	0.008	0.112		0.023	0.247	-0.009	0.179	0.054	0.046	0.066	
Stroop word	0.088	-0.024	0.043	-0.125	-0.174	0.025	0.063	-0.047		-0.051	-0.051	0.009	-0.002	0.211	0.176	0.138	
Stroop color	0.175	0.145	0.105	-0.110	-0.131	-0.010	0.115	0.049		0.097	0.089	0.119	-0.006	0.022	0.074	0.071	
Stroop color-word	0.188	0.107	0.049	0.021	-0.028	-0.085	0.014	-0.039		0.074	0.137	0.098	0.093	0.117	-0.019	-0.027	
Stroop total	0.182	0.085	0.083	-0.103	-0.153	-0.020	0.085	-0.017		0.039	0.058	0.087	0.028	0.157	0.114	0.090	

* *p*<0.05

** *p*<0.01

### Regression analysis on cognitive functions

For the unmedicated BD patients, TG was an independent contributor to language (β = 0.336, *p* = 0.010). And PRL was an independent contributor to the attention (β = 0.272, *p* = 0.039). Insulin was an independent contributor to Stroop word (β = -0.298, *p* = 0.023). Furthermore, AMH was an independent contributor to Stroop color (β = 0.266, *p* = 0.028) and Stroop total scores (β = 0.273, *p* = 0.026) (Supplementary Table S1).

For the stable-medicated BD patients, PRGE independently contributed to visuospatial/constructional (β = -0.299, *p* = 0.019) and Stroop color-word (β= -0.321, *p* = 0.009). And PRL was an independent contributor to attention (β = -0.373, *p* = 0.003) and Stroop color-word (β= -0.294, *p* = 0.015) (Supplementary Table S2).

For all BD patients, LDL-c was an independent contributor to Stroop color (β= -0.232, *p* = 0.009) and Stroop total scores (β =-0.214, *p* = 0.016). IRI was an independent contributor to Stroop word (β= -0.243, *p* = 0.008). And TST II was an independent contributor to immediate memory (β= -0.195, *p* = 0.033) (Supplementary Table S3).

## Discussion

Our study found abnormal alterations in glucolipids, reproductive hormone levels, and cognitive function, and correlations between glucolipids/reproductive hormones and cognitive dysfunction in female BD patients.

### Cognitive impairment in two female BD groups

We found that immediate memory, visuospatial/constructional, language, attention, delayed memory, Stroop word, Stroop color, Stroop color-word, Stroop total, and RBANS’ total scores were significantly lower in BD patients than in controls (*p* < 0.05), which are consistent with previous studies [29, 30]. These results suggested cognitive impairment in BD patients.

Some studies claimed that cognitive deficits may be rescued by medications, especially lithium. The maintenance of cognitive function in patients on long-term lithium treatment may be related to stimulation of the brain-derived neurotrophic factor (BDNF) system, thereby preventing affective episodes which have a deleterious effect on cognition [31]. Other drugs such as risperidone and quetiapine may cause side effects such as drowsiness, which may also be confused with BD-related neurocognitive deficits [32, 33]. However, medication was not found to cause effects on cognitive function in this study, which supports the notion that the impairment of cognitive function is the characteristics of BD. One study showed that high levels of peripheral inflammatory-cytokines, oxidative stress and reduced levels of BDNF were associated with poor cognitive performance, demonstrating a link between cognitive decline observed in BD and neuroinflammatory and neuroprotective mechanisms [34]. Other findings also linked cognitive impairment in BD to white matter changes in brain and to the morphological changes in the dorsomedial prefrontal cortex [35].

### Glucolipid, and reproductive hormone abnormalities in two female BD groups

Studies have shown a high prevalence of altered glycolipids in patients with mood disorders [36, 37]. In particular, women and patients with BD over 40 years of age are at particularly high risk [38]. Growing evidence showed that glycolipid disturbance is involved in the pathophysiology of BD [38–40]. However, our results revealed that there was only alteration of glycolipid in the stable-medicated patients, suggesting the effect of medication on glycolipid metabolism.

Many psychotropic drugs used to treat BD are associated with metabolic dysfunction, such as insulin resistance, and dyslipidemia [41]. And the metabolic dysfunction further exacerbates reproductive dysregulation in women with BD [42, 43]. It is also recognized that women with BD are prone to have reproductive dysfunction [43]. We only find reproductive alterations in stable-medicated patients, suggesting that reproductive dysfunction was induced by medication. Many drugs commonly used to treat women with BD have a deleterious effect on the hypothalamic pituitary-gonadal (HPG) axis [43]. The use of valproate (VPA) is associated with a high incidence of menstrual abnormalities (MA) and polycystic ovary syndrome (PCOS) [44], a poorly understood endocrine disorder. One previous study found that women with BD exhibited elevated LH: FSH, which may attribute to increasing LH secretion by VPA [41]. Consistent with previous study, we also found elevated LH levels in the stable-medicated group, confirming the influence of psychotropic medicine on reproductive function.

### Relationship between cognitive function and glucolipids/reproductive hormones in women with BD

Insulin resistance is emerging as a key factor in the neurological progression of BD [45]. And there is growing research evidence that central insulin resistance is associated with cognitive impairment in BD [46]. Previous studies also reported the association between lipids and cognition in BD patients. For example, elevated TG level was found to be related to executive function [47]. Decreased HDL-c was found to be associated with language and memory [19]. Our results also found that insulin resistance and lipid were related to decreased cognitive performance in patients with BD, which are in line with previous studies. We speculate that these correlations could be related to structural or functional alterations in brain regions due to lipid abnormalities. It was found that lipid profile abnormalities correlated with the magnitude of reduction in hippocampus [48, 49]. In the BD patients, decreased HDL-c was found to be associated with larger hippocampal volumes while elevated LDL-c and TG were associated with smaller hippocampal volumes [48].

Lipid abnormalities may contribute to cognitive impairment in BD through several pathways, and one possible mechanism is to affect the role of leptin. Leptin is a pleiotropic regulatory protein secreted by adipose tissue that crosses the blood-brain barrier (BBB) and acts on leptin receptors in brain to mediate many central nervous system (CNS) effects related to cognitive function [50]. It was found that TG in the cerebrospinal fluid can induce leptin resistance and insulin resistance [51]. One study also reported that TG may lead to changes in leptin dynamics by preventing leptin crossing the BBB [52]. Another possible pathological mechanism underlying the effects of lipids on cognition is oxidative stress and neuroinflammation. On the one hand, TG and their direct products can upregulate stress and inflammation-related gene expression, such as macrophage inflammatory protein 3α (MIP-3α), growth differentiation factor 15 (GDF15) and prostaglandin-endoperoxide synthase 2 (COX2) in brain [53]. On the other hand, elevated central inflammatory factors and activation of inflammatory cells may directly damage neuronal cells [54], all of which may lead to structural and functional changes in brain related to cognitive function in BD.

In our study, correlations between cognition and glycolipid/ reproductive hormones were observed in both unmedicated and medicated patients.

Several studies have mentioned the possible relationships between reproductive hormones and neuroprotection/neurodegeneration in women patients. First, estrogen and PRGE are involved in several aspects of brain function, such as brain development, synaptic plasticity, and regulation of neurotransmitters such as serotonin, norepinephrine, gamma-aminobutyric acid (GABA), and glutamate. Second, estrogen and PRGE receptors are present in brain regions associated with emotion regulation and cognition, including the hypothalamus, hippocampus, amygdala, and prefrontal cortex [55–58]. Finally, clinical data suggest that reproductive hormones affect certain mood and cognitive levels in women with BD and that there is a strong relationship between reproductive hormones and molecular markers of neuroprotection and neurodegeneration (e.g., BDNF, oxidative stress, and neuroinflammation) [59]. Two randomized, double-blind studies using the PRGE antagonist mifepristone to treat patients with BD found improvement of cognitive functioning, particularly in the areas of working memory and verbal learning [60, 61]. And Soria et al. also argued that reproductive hormone-related drugs are promising area of researches that may help improve cognitive outcomes in patients with BD [16].

### Limitation

First, this is a cross-sectional study, it is not yet possible to determine a causal link between abnormalities in glucolipids/reproductive hormones and cognitive impairment in women with BD. Therefore, a long follow-up of the three groups of female participants is necessary in the future. Second, the reproductive hormonal indicators we tested are only a fraction of all reproductive hormones in women. Future studies need to include more reproductive hormones for measurement to further explore the potential association between abnormal reproductive hormone levels and cognitive impairment in female BD patients. Finally, because the unmedicated and stable-medicated female BD patients we included in this study were relatively young, they are not well representative of the full age range of female BD patients. Therefore, in the future, wider age range appropriate adjustment of the age of female BD patients could improve the representativeness of women with BD.

## Conclusions

In conclusion, our results suggested that female BD patients existed abnormal glycolipid and reproductive hormone levels, and cognitive dysfunction. Abnormal glycolipid and reproductive hormone levels may be associated with cognitive deficits in female BD patients. Therefore, it is necessary to further investigate the mechanisms of glycolipid metabolism and reproductive hormonal abnormalities involved in cognitive function in female BD patients.

### Electronic supplementary material

Below is the link to the electronic supplementary material.

Supplementary Material 1

## Data Availability

The datasets used and analyzed during the current study are available from the corresponding authors on reasonable request.
