# Supplementary material for: Association among abnormal glycolipids, reproductive hormones, and cognitive dysfunction in female patients with bipolar disorder
Source: BMC Psychiatry. 2024 May 21;24:385. doi: 10.1186/s12888-024-05831-y (PMC11110249; doi:10.1186/s12888-024-05831-y)
Supplement: Supplementary file 1 — Supplementary Material 1 [file 12888_2024_5831_MOESM1_ESM.docx]

Supplementary Material

Table S1. The contribution of hormones and glycolipid to cognitive function in unmedicated BD patients

| BD(n=58) |  | Independent variable  β (*p)* | | | |  |  |
| --- | --- | --- | --- | --- | --- | --- | --- |
| Dependent variable | TG | PRL | AMH | Insulin | LDL-c | | |
| Immediate Memory | / | / | / | / | / | | |
| Visuospatial/Constructional | / | / | / | / | / | | |
| Language | 0.336(0.010) | / | / | / | / | | |
| Attention | / | 0.272(0.039) | / | /  / | / | | |
| Delayed memory | / | / | / | / | / | | |
| RBANS total | / | / | / | / | / | | |
| Word | /  / | / | / | -0.298(0.023) | / | | |
| Color | / | / | 0.266(0.028) | / | -0.389(0.002) | | |
| Color-word | / | / | / | / | -0.276(0.036) | | |
| Stroop total | / | / | 0.273(0.026) | / | -0.347(0.005) | | |

BD, bipolar disorder; TG, triglyceride; PRL, prolactin; AMH, anti-Müllerianhormone; LDL-c, low-density lipoprotein cholesterol; RBANS, the Repeatable Battery for the Assessment of Neuropsychological Status; Age, Education, BMI were controlled in the regression models for each dependent variable.

Table S2. The contribution of hormones and glycolipid to cognitive function in stable-medicated BD patients

| BD(n=61) | Independent variable  β (*p)* | | |
| --- | --- | --- | --- |
| Dependent variable | PRGE | PRL |  |
| Immediate Memory |  |  |  |
| Visuospatial/Constructional | -0.299(0.019) |  |  |
| Language |  |  |  |
| Attention |  | -0.373(0.003) |  |
| Delayed memory |  |  |  |
| RBANS total |  |  |  |
| Word |  |  |  |
| Color |  |  |  |
| Color-word | -0.321(0.009) | -0.294(0.015) |  |
| Stroop total |  |  |  |

BD, bipolar disorder; PRGE, progesterone; PRL, prolactin; RBANS, the Repeatable Battery for the Assessment of Neuropsychological Status; Age, Education, BMI were controlled in the regression models for each dependent variable.

Table S3. The contribution of hormones and glycolipid to cognitive function in all BD patients

| BD(n=119) | Independent variable  β (*p)* | | | |  |
| --- | --- | --- | --- | --- | --- |
| Dependent variable | | TST II | IRI | LDL-c | |
| Immediate Memory | | -0.195(0.033) |  |  | |
| Visuospatial/Constructional | |  |  |  | |
| Language | |  |  |  | |
| Attention | |  |  |  | |
| Delayed memory | |  |  |  | |
| RBANS total | |  |  |  | |
| Word | |  | -0.243(0.008) |  | |
| Color | |  |  | -0.232(0.009) | |
| Color-word | |  |  |  | |
| Stroop total | |  |  | -0.214(0.016) | |

BD, bipolar disorder; TST Ⅱ, testosterone Ⅱ; IRI, Insulin Resistance Index; LDL-c, low-density lipoprotein cholesterol; RBANS, the Repeatable Battery for the Assessment of Neuropsychological Status; Age, Education, BMI were controlled in the regression models for each dependent variable.
